# Evaluation of Population Pharmacokinetic Models of Micafungin: Implications for Dosing Regimen Optimization in Critically Ill Patients

**DOI:** 10.3390/pharmaceutics16091145

**Published:** 2024-08-29

**Authors:** Xiping Li, Xiaoqin Liu, Juehui Mao, Dong Liu, Zheng Jiao

**Affiliations:** 1 Department of Pharmacy, Tongji Hospital, Tongji Medical College, Huazhong University of Science and Technology, Wuhan 430030, China; lxp1987817@163.com (X.L.); ld2069@outlook.com (D.L.); 2 Department of Pharmacy, Shanghai Chest Hospital, Shanghai Jiao Tong University, Shanghai 200030, China; liuxq_1204@163.com (X.L.); 3321092062@stu.cpu.edu.cn (J.M.)

**Keywords:** micafungin, pharmacokinetic/pharmacodynamic, population pharmacokinetics, dosing regimen optimization

## Abstract

Micafungin (MFG) is a widely used echinocandin antifungal agent for treating invasive candidiasis, particularly in critically ill patients. However, its pharmacokinetics can be highly variable in this population. This systematic review aims to summarize population pharmacokinetic models and provide recommendations for its use in intensive care unit (ICU) patients. Monte Carlo simulations were implemented to compare pharmacokinetic parameters and probability of target attainment (PTA) against various *Candida* species. A total of 16 studies were included, of which 6 studies were conducted in adult ICU patients. The key covariates were body size, liver function, and sepsis-related organ failure assessment score (SOFA) score. The median MFG clearance in adult ICU patients was 30–51% higher than in adult non-ICU patients. For infections with *C. albican* with MIC below 0.016 mg/L, micafungin dosages of 100 and 150 mg/d were recommended for adult non-ICU and ICU patients, respectively. For *C. tropicalis* and *C. glabrata*, 200 and 250 mg/d were recommended, respectively. However, for *C. krusei* and *C. parapsilosis*, none of the tested dosage regimens achieved assumed PTA criteria within MIC ranges of 0.125–0.25 mg/L and 0.125–2 mg/L, respectively. Therefore, MFG dosage regimens in ICU and non-ICU patients should be tailored based on the *Candida* spp. and their respective MIC values.

## 1. Introduction

Micafungin (MFG) is a semisynthetic, high molecular weight, water-soluble echinocandin [1]. It exerts anti-*Candida* activity by non-competitively inhibiting the synthesis of 1,3-*β*-D-glucan within the cell wall. This inhibition may also prevent the growth of hyphae in *Aspergillus* species that are resistant to conventional antifungal agents [2]. Currently, this agent is licensed worldwide for the prophylaxis and treatment of invasive *Candida* infections [3,4].

Given its minimal absorption after oral administration, MFG is only available as an intravenous formulation. It has a high protein-binding rate (approximately 99.8%) and is rapidly distributed to tissues. Furthermore, it exhibits linear pharmacokinetics (PK) and can be used without dosage adjustment in patients with renal impairment and minor-to-moderate hepatic impairment. Moreover, no dose-limiting severe toxicity has been observed, even at dosages exceeding 8 mg/kg and 15 mg/kg for adults and neonates, respectively [5,6,7]. This favorable safety and efficacy profile renders MFG an alternative option for treating sensitive *Candida* infections in pediatric and adult patients.

However, there is significant PK variability and suboptimal exposure under standard MFG dosages, particularly in critically ill patients in the intensive care unit (ICU). Notably, the physiopathological conditions of these patients differ considerably from those of non-ICU patients, affecting the functions of important organs. ICU patients frequently require life-support equipment, including hemodialysis (HD), continuous renal replacement therapy (CRRT), or extracorporeal membrane oxygenation (ECMO). Increasing evidence suggests that treatment failure may result from fungal resistance and/or prolonged suboptimal drug exposure. Consequently, individualized dosage adjustment is of paramount importance in this population.

The clinical outcomes of MFG in treating invasive *Candida* infections are characterized by pharmacokinetic/pharmacodynamic (PK/PD) indices [8,9,10]. Theoretically, the ratio of the area under the plasma unbound drug concentration–time curve over 24 h to the minimum inhibitory concentration (*f*AUC_24_/MIC) at steady state should be a more suitable PK/PD indicator than the ratio of the area under the plasma total drug concentration–time curve over 24 h to the MIC (AUC_24_/MIC) [10]. However, the latter method is more commonly applied, particularly the percentage of time above the MIC (T > MIC %). The model proposed by Andes et al. [8] established a correlation between clinical and microbiological responses and determined the susceptibility cut-off targets of AUC_24_/MIC to be ≥5000 for *C. albicans* and *C. glabrata*, ≥3000 for *C. krusei* and *C. tropicalis*, and ≥285 for *C. parapsilosis* [8,11,12]. Enhancing the identification and utilization of factors influencing PK/PD is crucial for rationalizing and optimizing MFG dosing schedules. This systematic review aims to evaluate the quality of different models, summarize relevant covariates, and provide valuable recommendations for the rational use of MFG in clinical practice based on the probability of target attainment (PTA) range among various populations, particularly ICU patients, using the simulation results of the included models.

## 2. Materials and Methods

This systematic review was conducted according to the Preferred Reporting Items for Systematic reviews and Meta-Analyses (PRISMA) guidelines (see Supplementary Materials).

### 2.1. Search Strategy

Population pharmacokinetic (PPK) studies on MFG were systematically searched in PubMed, Embase, and Web of Science databases from their inception to 30 January 2023. Search terms included ‘FK 463’, ‘FK463’, ‘FK-463’, ‘Micafungin Sodium’, ‘Micafungin’, ‘Mycamine’ and ‘population pharmacokinetic*’, ‘pharmacokinetic model*’, ‘nonlinear mixed effect model’, ‘NONMEM’, ‘Pmetrics’, ‘WINNONMIX’, ‘ADAPT’, ‘P-PHARM’, ‘nlmixed’, ‘NLME’, ‘USC PACK’, and ‘MONOLIX’. The reference lists of included articles were also reviewed. The literature search was performed by two independent reviewers and inspected by a third author.

All published PPK models of MFG were included if they met the following criteria: (1) study population: patients or healthy individuals; (2) treatment with MFG as the study drug; and (3) data analysis: population PK or population PK/PD analysis.

Articles were excluded if they met the following criteria: (1) reviews, conference abstracts, or focused on methodology/algorithm/software; (2) non-English publications; (3) animal data only; (4) insufficient information on the methodology and PPK or PK/PD models.

### 2.2. Data Extraction

The following information was extracted from the identified studies: (1) characteristics and demographics of the study population (e.g., country/race, patients/healthy individuals, age, sex, body weight (BW), lean body weight (LBW), fat-free mass (FFM), ideal body weight (IBW); (2) study design (e.g., study type, number of included individuals and sampling, dosing regimens, and bio-assay method used); (3) modeling strategies and final parameter estimates (e.g., software/algorithm, structural model, statistical model, parameter estimates, covariates, between-subject variability (BSV), inter-occasion variability (IOV), and residual unexplained variability (RUV), model evaluation, and model application); (4) model application and recommended dosage regimens. The study characteristics and PPK analyses are summarized in a tabular format. 

### 2.3. Assessment of Literature Quality

According to the guidelines established by Kanji et al. [13] and Jamsen et al. [14], a 30-item checklist was used to evaluate the quality of the literature. The checklist ensures the inclusion of transparent and accurate clinical PK/PD studies. If an item in the checklist was reported in the study, one point was assigned; otherwise, no point was counted. The total score for each study was calculated and expressed as a percentage, which was defined as the compliance rate.

### 2.4. Comparison of Studies

#### 2.4.1. Assessment of Visual Predictive Distributions 

Visual predictive distributions (VPDs) of concentration–time profiles at steady state based on Monte Carlo simulations were performed in each eligible study [15]. According to the classifications of patient characteristics in the retrieved studies [16,17,18,19,20,21], we adapted the following 6 groups of virtual populations for Monte Carlo simulation: preschool children, school children, adolescents, and adults. The virtual adult population was divided into ICU (sepsis-related organ failure assessment score (SOFA) ≥ 10), ICU (SOFA < 10), and non-ICU groups. Additional details are provided in Table S1. MFG was intravenously administered over 1 h to all groups once daily for 7 days as follows: 4 mg/kg in neonates, 2 mg/kg in children younger than 12 years, and 100 mg in adolescents and adults. A steady state was assumed for all virtual populations. A total of 1000 virtual patients were simulated for each scenario. All simulations were performed using the NONMEM software (version 7.5; ICON Development Solutions, Ellicott City, MD, USA). The concentration–time profiles generated with different models were compared visually. Clearance (CL), central volume of distribution (Vd), and AUC_24_ were analyzed, also. 

#### 2.4.2. Assessment of the Covariates’ Impact

The effects of the included covariates on PK parameters were assessed using forest plots. For continuous covariates, the maximum and minimum values based on the demographic information in the included studies were extracted and scaled to the same range. Regarding binary covariates, each category was similarly disposed. In cases where one model used binary variables as covariates that could also apply to another model, corresponding cut-off values were used. Each binary and continuous covariate was input to explore its influence on the CL and Vd. The upper and lower limits of the parameters were estimated based on the range of the corresponding covariates and were normalized to the median values. Therefore, the effect of each covariate can be shown as the range of the limit to the median value as follows (Equation (1)):

(1) $$Effect\;of\;covariate\;(i)\;in\;model\;(j)=\frac{Estimated\;range\;of\;parameter\;(k)}{Median\;value\;of\;parameter\;(k)\;in\;model\;(j)}\times 100\%$$

We regarded covariate effects beyond the 80–125% range as clinically significant, according to the standards employed in previous studies [22,23,24]. All data were analyzed and plotted using R software (version 4.2.1; www.r-project.org, accessed on 30 January 2023).

### 2.5. Monte Carlo Simulation for the Probability of Target Attainment 

The cut-offs of PK/PD indices (AUC_24_/MIC) obtained from animal models have been extrapolated to humans [8]. The susceptibility cut-off targets of AUC_24_/MIC against various fungal strains provide a critical tool for optimal dosing strategies and definitions of clinically relevant drug resistance. These susceptibility cut-off targets of AUC_24_/MIC were defined as ≥5000 for *C. albicans* and *C. glabrata*, ≥3000 for *C. krusei* and *C. tropicalis*, and ≥285 for *C. parapsilosis.* The following MIC breakpoints were selected based on the EUCAST standard: *C. albicans* ≤ 0.016 mg/L, *C. glabrata* ≤ 0.032 mg/L, *C. krusei* ≤ 0.06 mg/L, *C. tropicalis* ≤ 0.25 mg/L, and *C. parapsilosis* ≤ 2 mg/L. The following CLSI standard MIC breakpoints were also considered: ≤0.25 mg/L for *C. albicans*, *C. krusei*, and *C. tropicalis*, ≤0.06 mg/L for *C. glabrata*, and ≤2 mg/L for *C. parapsilosis* [25,26]. 

Monte Carlo simulations were performed to explore the rationality of different dosing regimens in ICU and non-ICU adults. Six BW levels (40, 55, 70, 85, 115, 130 kg) with a fixed dose of 100 mg once daily and five dosage levels (100, 150, 200, 250, 300 mg, once daily) with a BW of 70 kg were investigated, respectively. One thousand virtual patients were simulated for each BW/dosage combination. The AUC_24_ on day 7 was calculated by dose/CL. The PTA analysis was determined by judging the probability of achieving adequate AUC_24_/MIC values against various *Candida* species. The optimal dosage for a particular *Candida* species is the minimum dosage that generates a PTA ≥ 90%.

## 3. Results

### 3.1. Study Identification

**Figure 1:**
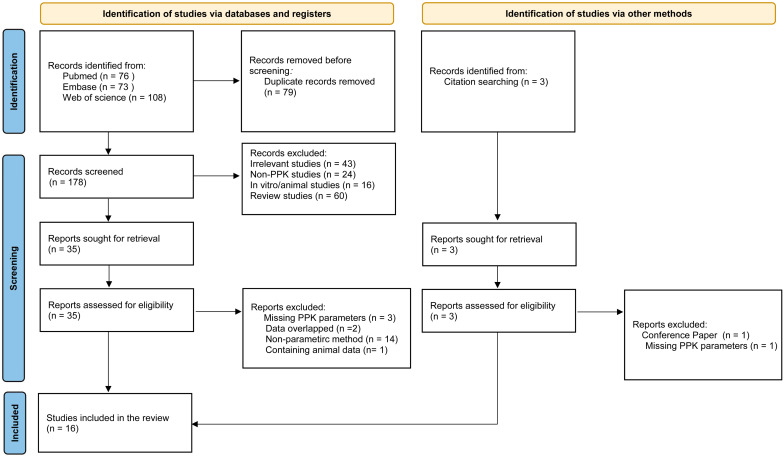
PRISMA flow diagram for identifying population pharmacokinetics studies of MFG.

A PRISMA diagram for this study is shown in Figure 1. A total of 76, 73, and 108 publications were initially selected from PubMed, EMBASE, and Web of Science, respectively. After excluding 79 duplicate records, 178 studies were included. A full-text review was conducted according to the screening criteria, and 14 studies were deemed eligible for inclusion. Additional studies were identified from the reference lists of included studies. Ultimately, 16 articles (17 models) published between 2006 and 2022 were retained.

### 3.2. Literature Quality

**Figure 2:**
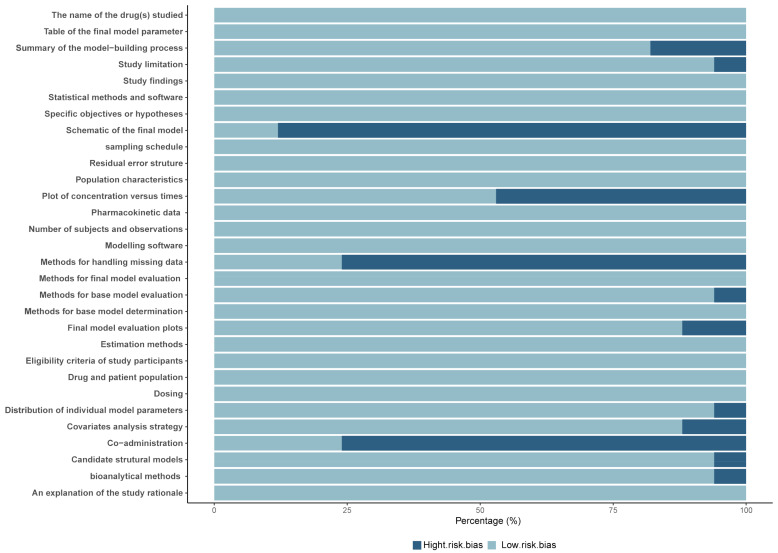
Risk bias map of the included population pharmacokinetics studies.

The 30-item checklist and the corresponding risk map of the studies are presented in Table S2 and Figure 2, respectively. Given that previous clinical studies identified that no potential drug–drug interactions altered the PK of MFG, most of the models (13/17) did not identify the use of concomitant medications as a covariate. A review of the model development sections of the 16 studies revealed that 13 studies lacked descriptions of the methods for handling missing data. Furthermore, only two studies included a schematic representation of the final model. Over 50% of the studies did not present a plot of concentration over time and/or the effect of concentration. The proportion of participants who adhered to the study protocol ranged from 76.7% to 96.7%, with a median adherence rate of 90%. All models, except one, achieved a compliance rate of at least 80%, indicating that these studies were of good quality. 

### 3.3. Study Comparison

The characteristics of all included studies are presented in Table 1. All studies were prospectively designed and published between 2006 and 2022. Two models involved healthy adult volunteers, whereas thirteen included adult patients. Four models were specific to pediatric populations: neonates (n = 1), infants (n = 4), preschoolers or schoolchildren (n = 4), and adolescents (n = 4). Seven models (41%) focused on a critically ill population. One of these models assessed the safety and PK of multiple elevated doses of MFG in preterm neonates. Over seven models (41%) were investigated for hematological malignancies, cancer, and hematopoietic stem cell transplantation. Nine models (53%) were developed using data from phase I, II, III, or IV clinical trials, whereas the remaining studies (47%) enrolled individuals from real-world clinical settings. The median number of participants was 24 (interquartile range (IQR): 13–54.8). Only 9 of 16 studies presented data on both BMI and height in conjunction with total body weight (TBW). The remaining seven studies provided only TBW. One study provided fat-free body weight (FFM) [27]. All studies acquired over four plasma samples per patient, except for one study, which did not accurately record the sample size [8].

The final PPK parameters of the included studies are presented in Table 2. All studies employed either a two-compartment model (n = 15) or a three-compartment model (n = 2), accompanied by zero-order infusion and first-order elimination, to describe the administration, distribution, and disposition procedure of MFG. MFG was administered over a wide range of infusion times (0.5–3 h) [16,18,28]. 

All studies explored BSV using the exponential model. The BSV values for CL and Vd were 24.1% coefficient of variation (CV) (IQR: 18.1–32.8%) and 34.3% CV (IQR: 16.7–51.6%), respectively. RUV was described using additive, proportional, or combined error models. Fourteen studies included proportional errors, with an IQR of 5.6–19% CV, and eight included additive errors, with an IQR of 0.0666–0.642 mg/L. The IOV was estimated to be 16.1% CV (IQR: 10–27.8%) for clearance (CL) and 27–28.1% CV for central volume of distribution (Vd).

In terms of model validation, 15 models (93.8%) were internally evaluated using more than two methods. However, none of these methods were validated using an external method. The most common validation methods included goodness-of-fit plots (GOF), visual predictive checks (VPC), and prediction-corrected VPC (pcVPC) checks. Although the normalized prediction distribution error (NPDE) method is an effective evaluation tool, it was employed in only two studies [12,29]. Ten out of the sixteen studies employed non-parametric bootstrap validations. 

Fifteen of the seventeen models (88%) conducted Monte Carlo simulations to validate the investigated dosages or propose new dose recommendations. The PK/PD indicators included AUC_24_/MIC (n = 9), *f*AUC_24_/MIC (n = 1), AUC_24_ (n = 4), and T > MIC (%) (n = 1). In the context of pediatric patients, five studies [16,18,19,20,21] not only supplemented the most recent labeled dosage regimens but also further validated the feasibility of an intermittent dosing strategy compared to a daily dosing strategy [18,19,21]. Ultimately, the studies concluded that a dose of 15 mg/kg/day in premature neonates nearly equaled 5 mg/kg/day in adults. Chandra et al. [19] proposed that 5 mg/kg twice weekly might be sufficient for pediatric patients with low BW (<30 kg) for fungal infection prophylaxis. Nevertheless, caution was advised for pediatric patients with a higher BW (≥30 kg). Didi et al. [18] concluded that alternative strategies for *Candida* prophylaxis could include dosing of 5, 7, 9 mg/kg twice weekly or a flat dosing approach by weight bands, categorized as follows: 100 mg for patients with a BW ≤ 20 kg, 150 mg for those with a BW of 20–40 kg, 300 mg for those with a BW ≥ 40 kg.

In 13 models of adult populations, 2 studies [17,28] demonstrated the rationalization of approved dosages for *Candida* infections. Muilwijk et al. [28] recommended extending the dosing interval to 300 mg once weekly (3 h infusion) for both the prophylaxis and treatment of *Candida* infections. Another study [17] proposed that a dosage of 200–250 mg/d should be initiated to enhance the likelihood of a favorable outcome for *Aspergillus* infections. In another study, Roeland et al. [27] indicated that the current standard dosage is insufficient for obese patients weighing ≥ 125 kg. Six studies [11,12,29,30,31,32] evaluated the PTA of various intermittent dosing scenarios versus daily dosing regimens in adult ICU patients. Among these, two studies [11,12] indicated that the current dosages were adequate to sustain a PTA ≥ 90% with an MIC not inferior to 0.016 mg/L. However, the daily dose should be increased to 200 mg with an MIC of 0.032 mg/L for adult ICU patients, regardless of *Candida* spp. Zhong et al. [29] indicated that the daily dose for ICU patients with a high SOFA score (>10) should be further increased to 250 and 300 mg to achieve PTA targets, with attenuated MICs of 0.032 and 0.064 mg/L for *C. glabrata* and *C. tropicalis*, respectively.

**Table 1:** Study characteristics.

Study (Year)	Study Type	Country /Race	Study Population	No. of Subjects (M/F)	No. of Samples (Per Person)	Age (Years) Mean ± SD Median [Range]	Body Weight (kg) Mean ± SD Median [Range]	Dosing Regimens	Bioanalytical Method [LLOQ, mg/L]
Kenji Tabata et al. (2006) [16]	Phase I, II, III	Japan	Healthy subjects	82	1353 (16.2)	1353 (16.2)	62.8 [45.1–80.6] ^a^	2.5–150 mg 12.5–150 mg 1–6 mg/kg	HPLC-FLD [0.05]
			Adult patients	97	395 (4.1)	395 (4.1)	50.3 [28–76.4] ^a^		
			Pediatric patients	19	77 (4)	77 (4)	22.0 ± 14.0 [7–48]		
Kazuro Ikawa et al. (2009) [17]	Prospective	Japan	Adult hematology patients	10 (4/6)	48 (4.8)	63.5 + 16.2 [30–79]	55.4 ± 10.3 [46.0–77.4]	50–300 mg, single dose	HPLC-FLD [0.05]
P.B. Smith et al. (2009) [21]	Phase I	America	Critically ill preterm neonates > 48 h	34 (21/13)	NA (>5)	GTA: 26.65 [23–39] ^c^ PCA: 30.45 [26–39] ^c^ PTA: 26.7 [2–82] ^a^	1.185 [0.54–2.2] ^a^	15 mg qd, 5 days 0.75 mg/kg, 1.5 mg/kg, 3.0 mg/kg, single dose	HPLC-MS/MS [0.05]
David Andes et al. (2011) [8]	Phase III	North America, Europe, Brazil, India, Thailand, South Africa, Australia	Invasive candidiasis or *candidemia* infection	493 (290/203)	NA	55 [13–89] ^b^	68 [28–155] ^b^	100–150 mg qd, 14–56 days	NA
Emilio Maseda et al. (2014) [30]	Prospective	Spain	ICU patients	10 (8/2)	280 (28)	72 ± 8.2 73.5 [54–83]	69.6 ± 6.3 70.0 [61–80]	100 mg qd	HPLC-UV [0.2]
William W. Hope et al. (2015) [20]	Phase I, II	America	Treatment or prophylaxis against *aspergillus* spp. or *candida* spp.	229	1919 (8.4)	0.3 to <2 years: 1.0 ± 0.4	7.9 ± 1.7	0.5, 1, 1.5, 2, 3, 4, 4.5 mg/kg qd	HPLC-FLD [0.05]
						2–5 years: 3.7 ±1.2	15.3 ± 4.4		
						6–11 years: 9.0 ± 1.5	28.9 ±9.0		
						12–16 years: 14.5 ±1.5	54.4 ± 17.3		
Lisa C. Martial et al. (2017) [11]	Prospective	America	ICU patients	20 (8/12)	356 (17.8)	68 [20–84]	76.5 [50–134]	100 mg qd	HPLC-UV [0.01]
Vincent Jullien et al. (2017) [12]	Phase III	France	ICU patients	99 (66/33)	436 (4.4)	61.4 [29.9–92.7]	84.5 [48–141]	100 mg qd, 14 days	HPLC-FLD [0.2]
E. W. Muilwijk et al. (2018) [28]	Phase II	The Netherlands	Adult hematology patients	20 (12/8)	~340 (17)	59.5 [38–68]	86.6 [53.5–110.1]	300 mg twice a week or 100 mg qd	HPLC-FLD [0.01]
Sharat Chandra et al. (2018) [19]	Phase I	America	HSCT patients	24 (6/18)	267 (11.1)	3.8 [0.6–10.4]	15.4 [7.7–30.3]	5 mg/kg, every 4 days	HPLC-UV [0.05]
Roeland E. Wasmann et al. (2019) [27]	Phase IV	The Netherlands	Healthy volunteers or obese adults	24 (12/12)	~240 (10)	31 [22–56] ^d^ 51 [35–61] ^e^ 46 [24–54] ^f^	70.8 [61.5–81.5] ^d^ 156 [112–184] ^e^ 141 [126–180] ^f^	Morbidly obese subjects: 100 mg or 200 mg Normal-weight subjects: 100 mg	UPLC-FLD [0.01]
Silke Gastine et al. (2019) [31]	Prospective	Germany	Critically ill patients	36 (24/12)	NA (≥9)	65 [22–84]	94.5 [49.9–162]	100 mg qd	HPLC-FLD [0.1]
Zhong Shubai et al. (2019) [29]	Prospective	China	Sepsis patients	32 (21/11)	153 (4.8)	60.1 [23.0–89.0] ^a^	70.22 ^a^ [55.0–90.0]	100, 150, 200 mg qd	HPLC-UV [0.2]
Iasonas Kapralos et al. (2020) [32]	Prospective	Greece	Critically ill patients	14 (7/7)	210 (15)	61 ± 15 [31–83]	85 ± 22 [55–130]	100 mg qd	HPLC-FLD [0.059]
Saeed Alqahtani et al. (2021a) [33]	Prospective	Saudi Arabia	Noncancer patients	9 (6/3)	63 (7)	51.1 ± 19.1	69.8 ± 15.7	100–150 mg qd, two doses	HPLC-UV [0.1]
Saeed Alqahtani et al. (2021b) [33]	Prospective	Saudi Arabia	Cancer patients	10 (6/4)	70 (7)	47.3 ± 12.3	63.4 ± 18.2	100 mg qd, two doses	HPLC-UV [0.1]
Didi Bury et al. (2022) [18]	Phase IV	The Netherlands	Pediatric patients	61 (34/27)	~420 (>5)	4.0 [1.0–17]	19.5 [8.60–182]	9 mg/kg (maximum 300 mg), twice a week	UPLC-FLD [0.01]

^a^ these data are listed as mean or average ± standard deviation [min–max]; ^b^ these data are listed as median [min–max]; ^c^ the unit is week; ^d^ classified as group of patients in normal weight administered with 100 mg micafungin; ^e,f^ classified as group of patients in obese weight administered with 100 mg, 200 mg micafungin, respectively; PCA/weeks, Postconceptional age; GTA/weeks, Gestational age; PTA/days, Pregnancy termination age.

**Table 2:** Final population pharmacokinetic parameters of included studies.

Study (Year)	Software/ Algorithm	Compartment	Fixed Effect Parameters		Between Subject Variability	Residual Unexplained Variability	Model Evaluation	Model Application
Kenji Tabata et al. (2006) [16]	NONMEM FOCE-I	2 CMT zero-order input first-order elimination	CL (mL/min)	13.0 + 0.228 × (BW-2.3) × FIX + 0.0345 × (PLT-21.6) (IF AGE ≥ 16, FIX = 0, IF AGE < 16, FIX = 1)	23.80%	11.00%	GOF; VPC	NA
			V (L)	11.2	23.80%			
			Vss (L)	20.6	23.80%			
			Q (mL/min)	96.5	23.80%			
Kazuro Ikawa et al. (2009) [17]	NONMEM FOCE-I	2 CMT zero-order input first-order elimination	CL (L/h)	0.762	15.40%	0.642 mg/L	GOF, bootstrap	Assessment of micafungin regimens based on PTA of fAUC_24_/MIC against Aspergillus
			Vd (L)	9.25	24.60%			
			Vp (L)	8.86	71.80%			
			Q (L/h)	7.02	0 FIXED			
P Brian Smith et al. (2009) [21]	NONMEM FOCE	2 CMT zero-order input first-order elimination	CL (L/h)	0.0365	48.80%	29.20%	NA	NA
			V (L)	0.507	48.80%			
			Vss (L)	1.6	48.80%			
			Q (L/h)	0.0316	/			
David Andes et al. (2011) [8]	NONMEM FOCE-I	2 CMT zero-order input first-order elimination	CL (L/h)	1.05 × (BW/65)^0.258^	36.00%	19.30%	GOF	Explore the relationship between clinical outcome and microbiological response.
			Vd (L)	10.2	28.30%			
			Vp (L)	10.3	50.50%			
			Q (L/h)	6.59	84.50%			
Emilio Maseda et al. (2014) [30]	NONMEM FOCE-I	2 CMT zero-order input first-order elimination	CL (L/h)	0.88 × (BW/70)^0.75^	20.20%	1.30% 0.36 mg/L	GOF, bootstrap, VPC	Evaluate covariate effects; Describe PK in specific populations.
					22.1% (IOV)			
			Vd (L)	12.5	8.30%			
					28.1% (IOV)			
			Vp (L)	10	7.50%			
					27.4% (IOV)			
			Q (L/h)	5.03	/			
William W. Hope et al. (2015) [20]	NONMEM FOCE-I	2 CMT zero-order input first-order elimination	CL (L/h)	0.356 × (BW/21.5)^0.787^ × (AST/50)^−0.0601^ × (TBIL/12)^−0.0492^	28.90%	17.69% 35.92%^a^ 0.0666 mg/L	GOF, bootstrap	Evaluate covariate effects; Describe PK in specific populations; Identify therapeutic micafungin regimens.
			Vd (L)	1.21	98.30%			
				4.62	16.61%			
			Q (L/h)	5.54	123.20%			
Lisa C. Martial et al. (2017) [11]	NONMEM FOCE-I	2 CMT zero-order input first-order elimination	CL (L/h)	1.1	40.10%	17%	GOF, bootstrap, pcVPC	Evaluate covariate effects; Optimize dosing regimens.
			Vd (L)	17.6	73.20%			
			Vp (L)	3.63	37.0% (IOV)			
			Q (L/h)	0.363	/			
Vincent Jullien et al. (2017) [12]	NONMEM FOCE-I	2 CMT zero-order input first-order elimination	CL (L/h)	1.34 × (BW/84)^0.59^ × 1.14 (if ALB ≤ 25 g/L) × 0.75 (if SOFA ≥ 10)	11.40%	1.44%	GOF, bootstrap, VPC, NPDE	Evaluate covariate effects; Analyze the PK/PD in specific populations; Evaluate the PTA of dosing regimens; Optimize dosing regimens.
			Vd (L)	11.8 × (BW/84)^0.61^ × 1.14 (if ALB ≤ 25 g/L)	37.81%			
			Vp (L)	7.68 × (BW/84)^0.67^ × 1.14 (if ALB ≤ 25 g/L)	15.00%			
			Q(L/h)	4.67	13.90%			
EW Muilwijk et al. (2018) [28]	NONMEM FOCE-I	3 CMT zero-order input first-order elimination	CL (L/h)	1.01 × (FFM/57.18)^0.75^	21.30%	7.71% 0.0878 mg/L	GOF, bootstrap, VPC	Evaluate the PK rationale of extending the dosing interval in special populations.
					9.78% (IOV)			
			V1 (L)	6.26 × (FFM/57.18)^1^	48.10%			
			V2 (L)	6.26 × (FFM/57.18)^1^	48.10%			
			V3 (L)	6.26 × (FFM/57.18)^1^	48.10%			
					0.809 ^b^			
			Q1 (L/h)	10.3 × (FFM/57.18)^0.75^	/			
			Q2 (L/h)	2.04 × (FFM/57.18)^0.75^	/			
Sharat Chandra et al. (2018) [19]	NONMEM FOCE-I	2 CMT zero-order input first-order elimination	CL (L/h)	0.78 × (BW/70)^0.75^	20.50%	18% 0.15 mg/L	GOF, pcVPC, bootstrap	Describe PK in specific populations; Evaluated the PK rationale of extending the dosing interval of micafungin.
			Vd (L)	13.9 × (BW/70)	31.20%			
			Vp (L)	5.9 × (BW/70)	0			
			Q (L/h)	1.1 × (BW/70)^0.75^	78.30%			
Roeland E. Wasmann et al. (2019) [27]	NONMEM FOCE-I	2 CMT zero-order input first-order elimination	CL (L/h)	0.690 × (BW/70)^0.74^	8.10%	5%	GOF, pcVPC, bootstrap	Evaluate covariate effects; Describe PK; Optimize dosing regimens in special populations.
			Vd (L)	5.84 × (BW/70)^1.17^	12.80%			
			Vp (L)	6.96 × (BW/70)^0.71^	/			
			Q (L/h)	7.15	/			
Silke Gastine et al. (2019) [31]	NONMEM FOCE-I	2 CMT zero-order input first-order elimination	CL (L/h)	1.56 × 0.789 (IF TBIL > 4 mg/dL)	48.90%	0.26%	GOF, VPC	Evaluate covariate effects; Describe PK in specific populations; Evaluate the efficacy of dosing regimen.
			Vd (L)	16.2 × 0.692 (IF SOFA > 10)	70%			
			Vp (L)	13.8	/			
			Q (L/h)	14.4	/			
Iasonas Kapralos et al. (2020) [32]	NONMEM FOCE-I	2 CMT zero-order input first-order elimination	CL (L/h)	1.31	19.00%	14.90%	GOF, bootstrap, pcVPC	Analyze the PK/PD in specific populations; Optimize dosage regimens.
					45% (IOV)			
			Vd (L)	14.2	18.00%			
					27% (IOV)			
			Vp (L)	12.6	51.00%			
			Q (L/h)	2.89	63.00%			
Zhong Shubai et al. (2021) [29]	NONMEM FOCE-I	2 CMT zero-order input first-order elimination	CL (L/h)	0.76 × e^((ALT/43) × (−0.268))^	24.10%	1.06 mg/L	GOF, VPC, bootstrap, NPDE	Evaluate covariate effects; Evaluate the PK rationale for extending the dosing interval.
			Vd (L)	6.7	52.80%			
			Vp (L)	10.2 × e^(θ × (−1.08))^ (SOFA < 10, θ = 0; SOFA ≥ 10, θ = 1)	78.87%			
			Q (L/h)	4.72	/			
Saeed Alqahtani et al. (2021a) [33]	Monolix SAEM	2 CMT zero-order input first-order elimination	CL (L/h)	0.6	11.80%	38.70% 0.42 mg/L	GOF, pcVPC	Describe PK; Analyze the PK/PD in specific populations; Evaluate the PTA of different dosing regimens with or without cancer.
			Vd (L)	12	7.60%			
			Vp (L)	2.77	20.40%			
			Q (L/h)	0.188	32.10%			
Saeed Alqahtani et al. (2021b) [33]	Monolix SAEM	2 CMT zero-order input first-order elimination	CL (L/h)	1.2	34.10%	45.82% 0.47 mg/L	GOF, pcVPC	Describe PK; Analyze the PK/PD in specific populations; Evaluate the PTA of different dosing regimens with or without cancer.
			Vd (L)	10.7	7.60%			
			Vp (L)	3.5	36.80%			
			Q (L/h)	0.144	32.20%			
Didi Bury et al. (2022) [18]	NONMEM FOCE	2 CMT zero-order input first-order elimination	CL (L/h)	0.678×(FFM/57.19)^0.75^	24.90%	9%	GOF, pcVPC	Evaluate the PK rationale for extending the dosing interval in special populations.

^a,b^ Different proportional error applied to patients (n = 25) in study 9463-CL-2101 with highly variable trough screen data; CMT, compartment; Vss, volume of distribution at steady-state; V1, central volume; V2, V3, peripheral 1 or 2 volume; Q, Q1, or Q2, intercompartmental 1 or 2 clearance; Free-drug AUC/MIC over 24 h, *f*AUC_24_/MIC; FOCE, first-order conditional estimation; FOCE-I, first-order conditional estimation with interaction; SAEM, stochastic approximation expectation maximization algorithm.

### 3.4. Visual Predictive Distributions 

**Figure 3:**
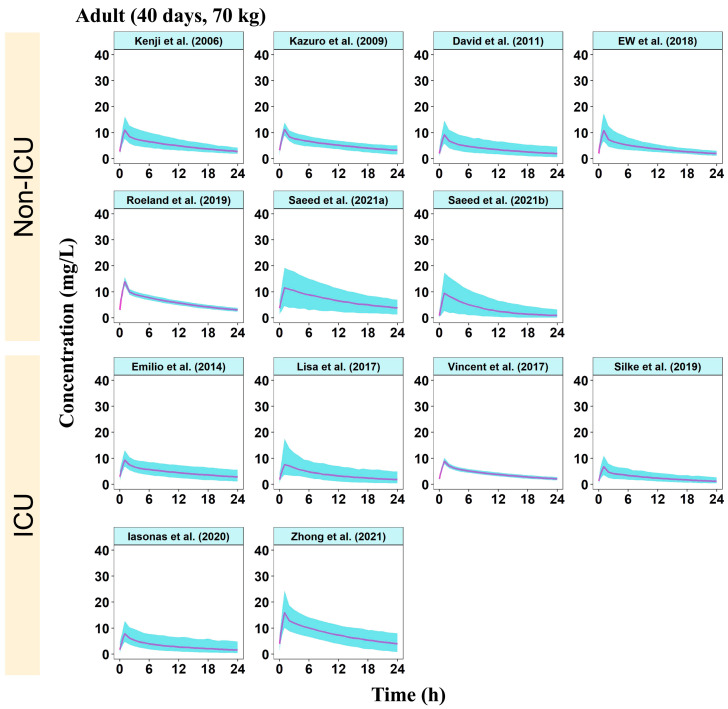
Simulated concentration–time profiles of MFG over 24 h at steady state in 70-kg adult patients administered with multiple doses of 100 mg MFG once daily [8,11,12,16,17,27,28,29,30,31,32,33]. The solid purple lines represent the median of the simulated concentration–time profiles and the light blue shadows represent the 5th–95th percentiles of the concentration–time profiles. MFG, micafungin.

The concentration–time profiles of MFG in the different virtual populations of pediatric and adult patients are shown in Figure S1 and Figure 3, respectively. The model established by Smith et al. [21] demonstrated a greater BSV than any other pediatric subgroup. This may be attributed to the low BW and relatively small sample size, with the added complexity of sourcing samples from critically ill pediatric patients, further amplifying the variability. The inclusion of only one neonatal model precluded intragroup comparison. Second, for children older than four months, the model established by Didi et al. [18] exhibited higher peak concentrations of MFG than the other models within each pediatric group. For adults, all included models displayed similar PK profiles, except for the model by Zhong et al. (2021) [29], which showed higher peak and trough drug concentrations than the other models.

### 3.5. Pharmacokinetic Parameters

**Figure 4:**
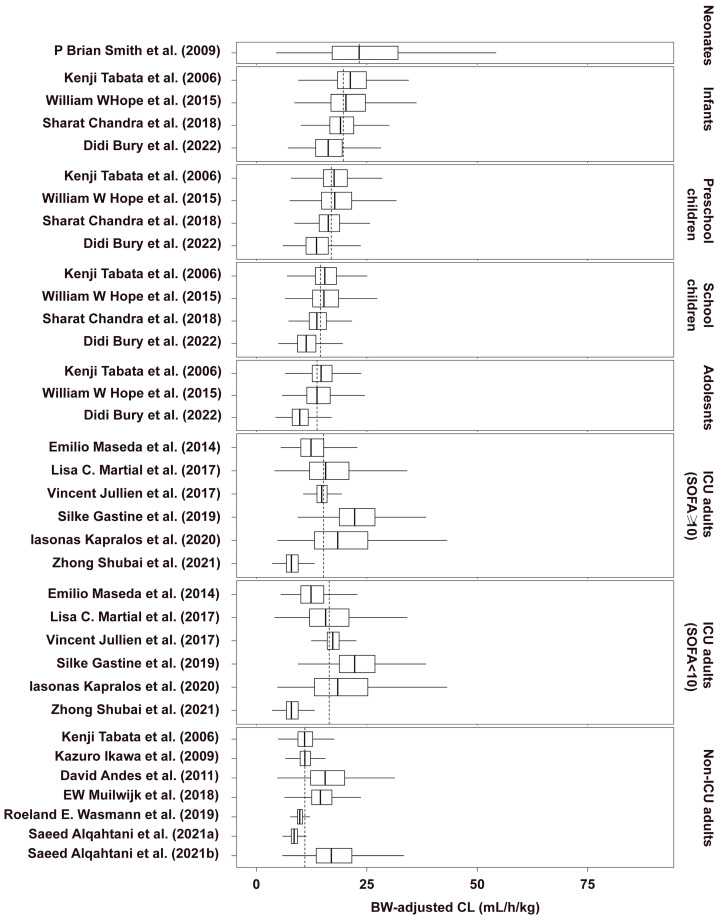
Distribution of the BW-adjusted CL of micafungin at steady state for various typical populations [8,11,12,16,17,18,19,20,21,27,28,29,30,31,32,33]. The SOFA scores were set to 7 for ICU patients with SOFA < 10 and 11 for ICU patients with SOFA ≥ 10. The vertical dashed lines in each panel represented the median values of CL per body weight from all patients within each group.

A comprehensive comparison of the simulated CL and Vd of MFG at steady state is shown in Figure 4 and Figure S2, respectively, and the results of PK parameters for various groups are listed in Table 3.

Except for adolescents, significantly lower MFG CL and Vd levels were observed in the other pediatric groups than in non-ICU adult patients. However, the ratios of the estimated median CL values to per unit BW in neonates, infants, preschool children, school children, and adolescents to non-ICU adults were 2.05, 1.71, 1.44, 1.23, and 1.13, respectively. For the ICU population, a notable heterogeneity in PK parameters was found. The median values of CL for ICU patients with SOFA scores ≥ 10 (0.75–1.38 L/h) and <10 (0.75–1.41 L/h) were 30% and 40% higher, respectively, than those for non-ICU adults. After excluding the only study conducted on a Chinese population [29], the CL values for ICU adults with SOFA scores ≥ 10 (0.91–1.48 L/h) and <10 (0.94–1.49 L/h) were 40% and 51% higher, respectively, than for non-ICU adults. This complicates the interpretation of the clinical differences in PK parameters between ICU and non-ICU groups, as the heterogeneity among ICU patients may be influenced by various factors not accounted for in the comparison.

### 3.6. Covariate Effect on Pharmacokinetic Parameters

**Figure 5:**
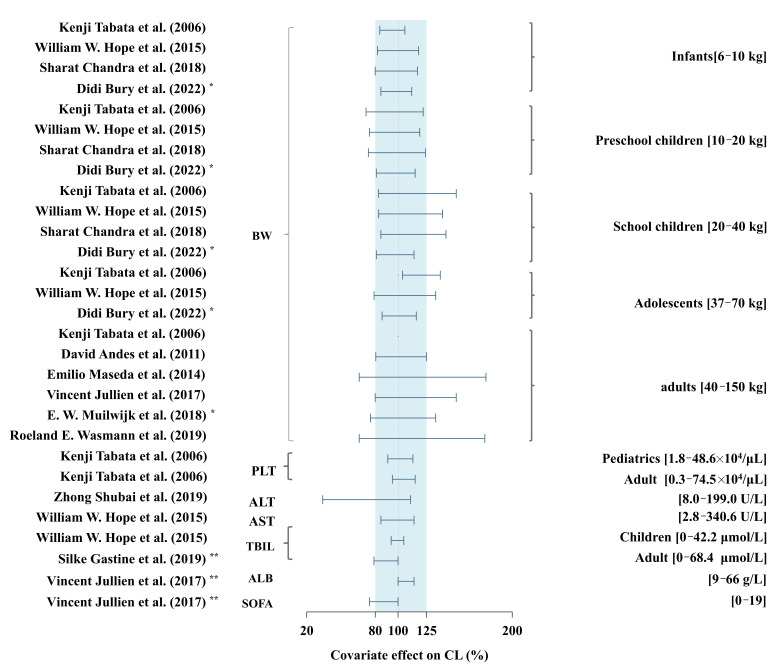
The effect of covariates on CL of micafungin in included studies [8,12,16,18,19,20,27,28,29,30,31]. * BW was transformed to equivalent FFM; ** For binary covariates, SOFA, 0 for SOFA ≥ 10 and 1 for SOFA < 10; TBIL, 0 for TBIL ≥ 68.4 μmol/L and 1 for TBIL < 68.4 μmol/L; ALB, 0 for ALB ≤ 25 (g/L) and ALB > 25 (g/L); BW, body weight; FFM, free-fat mass; ALT, alanine amino transferase; AST, aspartate aminotransferase; PLT, platelet count; TBILI, total bilirubin; ALB, albumin; SOFA, sepsis-related organ failure assessment score.

The characteristics of covariate screening and covariate-effect values are illustrated in Figure S3, Table S3 and Table S4, respectively. A total of nine studies identified body size as a crucial covariate on both CL and Vd. The effect of identified covariates on CL is presented in the forest map (Figure 5). Seven studies confirmed that the impact of BW or FFM on CL exceeded the assumed range (80–125%). Among all pediatric studies, the number of studies in which BW brings over 20% of changes for CL was as follows: preschool children (n = 3), schoolchildren (n = 3), and adolescents (n = 2). Four adult studies indicated that BW had a significant influence on CL, among which the greatest change was almost 80%, as reported by Roeland et al. [27] and Emilio et al. [30]. Two studies [29,31] showed that alanine aminotransferase (ALT) and total bilirubin (TBIL) levels within the normal ranges affected MFG clearance by more than 20%. Only one study confirmed the SOFA score as a covariate for CL. Compared with the reference value, the impact of the SOFA score on CL exceeded 20%.

### 3.7. Analysis of Probability of Target Attainment

**Figure 6:**
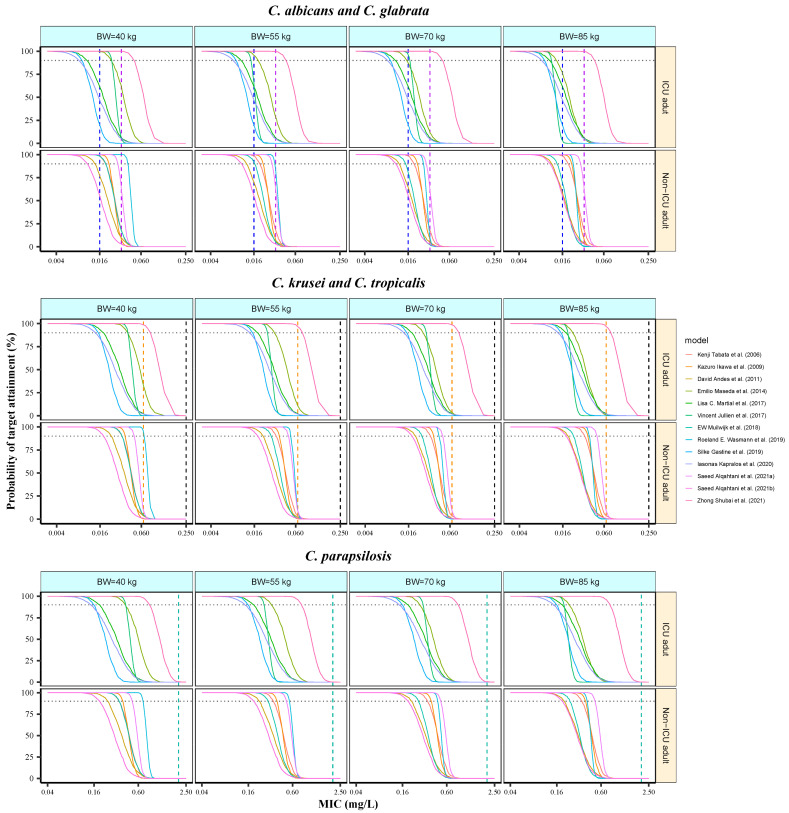
The PTA of micafungin for ICU adults or non-ICU adults against *Candida* spp. over 24 h at steady state [8,11,12,16,17,27,28,29,30,31,32,33]. The MIC breakpoints for *C. albicans* (blue), *C. glabrata* (purple), *C. krusei* (black), *C. tropicalis* (orange), and *C. parapsilosis* (green) are marked vertically with dashed lines in each panel, respectively. A PTA of 90% is highlighted horizontally with black dashed lines. All patients were administered intravenously with 100 mg MFG once daily for 7 days. The SOFA score in the ICU group was set to 11.

The PTA of MFG was simulated to assess its feasibility (Figure 6 and Figure S4). In non-ICU adults with low BW, the simulated exposure indicated that a PTA of ≥90% could be reached in most studies (5/7) at clinical MIC breakpoints for *C. albicans*, and even at BW increased up to 130 kg, there were still 4/7 studies maintaining the PTA targets. However, in the ICU group, 2/6 studies reached a PTA of ≥90% under similar simulation conditions, and if BW exceeded 70 kg, only 1 study met the PTA criteria. For *C. glabrata* (MIC = 0.032 mg/L), except for one study from the ICU group, none of the remaining studies from the ICU or non-ICU group achieved the assumed PTA criteria.

For *C. glabrata* (MIC = 0.032 mg/L), none of the studies from either the ICU or non-ICU groups achieved the target PTA, except for one model from the ICU group. For *C. krusei* (MIC = 0.25 mg/L) and *C. tropicalis* (MIC = 0.064 mg/L), none of the studies, excluding one from either group with low BW, met the PTA criteria. For *C. parapsilosis* (MIC = 2 mg/L), no studies achieved a PTA of ≥90%. According to CLSI standard, a 100 mg dose did not achieve a PTA of ≥90% for any of the *Candida* spp. tested.

The minimum dosage to sustain given ratios of studies achieving optimal dosage criteria is presented in Table 4. The simulated results indicated that 100–150 mg/day and 150–200 mg/day of MFG provided relatively adequate exposure for non-ICU and ICU adults, respectively, for *C. albicans*. For *C. tropicalis* and C. glabrata, recommended doses were 200–300 mg/day and ≥250 mg/day, respectively. However, even a high dose of 300 mg/day did not achieve satisfactory PTA for MICs of 0.125–0.25 mg/L for C. *krusei* and 1–2 mg/L for *C. parapsilosis*.

## 4. Discussion

MFG is a potent echinocandin employed to prevent and treat invasive infections caused by *Candida* and *Aspergillus*. Numerous studies have investigated its PK profiles, and several studies have explored the physiopathological factors affecting exposure variability. To date, this study is the first to systematically summarize the characteristic features of PPK modeling for MFG and to further explore whether dosing regimens for MFG should be adjusted for adult ICU patients. Most current studies indicate that age, body size, liver function, and SOFA score are the primary sources of PK variability. Moreover, multiple models have corroborated the rationale for extending dosing intervals based on PK principles. Finally, it was determined that an increase in the maintenance dose by 30–51% was necessary for adult ICU patients. Additional dose adjustments were recommended following the assessment of BW, SOFA score, and cultured MICs.

BW accounted for a significant proportion of the variability in CL and Vd of MFG. The findings of our study indicate that pediatric patients exhibited lower CL and Vd values than adults. However, CL standardized to allometrically scaled weight is constant in children. Furthermore, the increased BW-adjusted CL and Vd of MFG gradually decreased with age or BW, eventually reaching a plateau comparable to that observed in adults until children exceeded 8 to 9 years of age or reached a BW of 40–50 kg. This finding aligns with the conclusions of Seibel et al. [34] and Hope et al. [20]. This age difference may be attributed to several physiopathological factors, including reduced blood flow, altered body fat-to-lean mass ratio, and decreased total body water associated with metabolic maturation. Compared to adults, pediatric patients exhibit distinct physiological and pathological characteristics, as well as differences in drug-handling processes, which result in substantial PK disparities. This divergence is pronounced in infants whose renal excretion and hepatic metabolism are not yet fully mature. These factors significantly influenced the absorption, distribution, metabolism, and excretion of MFG. These findings theoretically justify the rationale behind the dosage recommendations from approved manufacturing labels. Nevertheless, further studies are necessary to determine optimal dosing strategies for neonates.

MFG clearance was significantly higher in obese adults. The relationship between CL and BW in this population is similar to that observed in healthy, normal-weight individuals [35,36]. A BW of 150 kg almost accounted for a change of 80% for CL (Figure 5). This is consistent with our simulation results (Figure S4), which showed a dosage of 100 mg/day is notably associated with reduced studies reaching the PTA criterion in the virtual population with a BW ≥ 90 kg, especially in the ICU group. These findings supported the opinion that the daily dose should be increased for obese adults. Furthermore, MFG is primarily metabolized by arylsulfatase, catechol-*O*-methyltransferase, and cytochrome P450 in the liver [37]. The correlation between CL and BW in obese individuals may be attributed to elevated cardiac output, liver blood flow, liver size, and the potential upregulation of arylsulfatase, which primarily participates in the metabolism of sulfate-containing lipids and may be more abundant in obese individuals.

Six studies consistently indicated that adult ICU patients exhibit higher clearance rates than non-ICU adults, suggesting a need to increase the loading or maintenance dose. However, there was substantial heterogeneity among these studies because of large variations. This variability may be attributed to the sample size and capricious physiological and pathological factors among the different cohorts. However, the study with the largest variation in the ICU group was based on a Chinese population, whereas the remaining studies were from other races, suggesting ethnic differences in the disposition of MFG. Unfortunately, statistical comparisons among races have been insufficient.

The clearance rate of MFG in ICU patients was unaffected by CRRT or IHD. This can be explained by the high binding affinity of MFG to ALB, which prevents its passage through the filter pores during RRT. This hypothesis is consistent with previous studies that used different RRT systems [38,39]. Consequently, dosage adjustments were not required for patients undergoing CRRT or IHD. The observed variations in CL and Vd among ICU patients between different dates were primarily attributed to hemodynamic instability. One study reported that ICU patients undergoing continuous venovenous hemofiltration exhibited lower inter-individual variability in the clearance and volume of distribution [11]. This discrepancy may be due to the more stable hemodynamics of patients undergoing CVVH, who are generally better managed for fluid retention and diuresis.

Our study also confirmed that the SOFA score has a significant impact on CL, reflecting a broader range of PK variability than any single liver function indicator such as TIBL, ALT, and AST. This is not contradictory with findings that ALT [29], AST [20], and TBIL [20,31] are covariates for CL. Sepsis-induced liver damage, whether through hemodynamic changes or direct hepatocyte injury, may affect liver function markers, but these markers alone may not fully capture the impact of sepsis. Specifically, a TBIL level > 4 mg/dL decreased CL by 21.1%, while a SOFA score > 10 reduced Vd by 30%, highlighting the comprehensive role of the SOFA score in assessing PK variability. These findings indicate that although routine dosage adjustment for MFG in mild-to-moderate liver dysfunction is not required, dosage adjustment should be considered for ICU patients with severe liver dysfunction (e.g., TBIL > 4 mg/dL). Accounting for ethnic differences, our study indicates that adult ICU patients have a 30–51% higher clearance rate compared to adult non-ICU patients, suggesting that maintenance doses should be increased by 30–51% for ICU patients. Further dose adjustments should be based on BW, SOFA score, liver function, and cultured MIC.

Furthermore, albumin levels had a minimal effect on the efficacy of MFG. This conclusion is supported by two key observations. First, half of the included studies evaluated the covariate effects of ALB; however, only one study identified and confirmed its impact on the PK parameters of MFG. This study demonstrated that the impact of ALB on either clearance or Vd was within 14%, a range that might not exert a substantial influence on its exposure. Second, ALB serves as a marker for protein binding. Hypoalbuminemia, which is commonly caused by liver cell injury, systemic inflammation, nephritic syndrome, and malnutrition, has long been a source of perplexity for experts who believe that it might increase the unbound drug concentration, leading to enhanced efficacy and potential risk of increased adverse reactions [40,41]. However, due to the high protein-binding rate and low hepatic extraction ratio [42], the percentage of unbound drugs is determined by the maximum binding capacity (B_max_) and equilibrium dissociation constant (K_D_) between the drug molecule and ALB [43]. The unbound MFG concentration remained unaltered in the presence of altered ALB levels. Only one study [29] identified ALB as a covariate of MFG CL; however, most studies employed PK/PD indicators based on total plasma concentration, which are only surrogate markers. Consequently, there was no need to modify the dosage in patients with hypoalbuminemia.

The dosage adjustment recommendations for different *Candida* spp. in our study were based on simulation results using the EUCAST standard. The clinical relevance of the difference in MIC cut-off values between the EUCAST and CLSI breakpoints remains unknown because the EUCAST breakpoints are slightly lower than their CLSI counterparts. For *C. albicans* at MICs of 0.016 mg/L, we recommend 100–150 mg and 150–200 mg/d of MFG for adult non-ICU and ICU patients, respectively. For *C. tropicalis* and *C. glabrata*, at MICs below EUCAST breakpoints, we recommended 200–300 mg/d and ≥250 mg/d, respectively. According to China-SCAN research, non-*Candida albicans* are more prevalent than *C. albicans* among ICU patients in China [44], suggesting the need for empirically higher initial doses to improve clinical outcomes in this special population before microbiological MICs are available. The simulation dosages did not achieve the assumed PTA targets for *C. krusei* with an MIC of 0.125–0.25 mg/L and *C. parapsilosis* with an MIC of 0.125–2 mg/L, indicating that antifungal agents, such as azoles and amphotericin B, may be more effective. These findings will aid in optimizing MFG dosing for various infections caused by *Candida* spp. in ICU patients.

This study has several limitations. First, plasma exposure is a surrogate marker for the infection site. It cannot be assumed that treatment failure would occur based on PTA targets calculated by combining the drug concentration levels over time in the central compartment and microbiological MICs. Second, only one model established the covariate effects of the SOFA score on CL, weakening the reliability of the conclusion. Therefore, it is necessary to verify relevant dosing recommendations in clinical cohorts. Moreover, further studies are necessary to validate dosing recommendations for neonates.

## 5. Conclusions

This study identified BW, liver function, and SOFA score as the primary factors influencing PK variation in MFG. One of the most significant findings of our study is the evidence that a 30–51% increase in MFG dose was required for ICU patients compared with non-ICU patients. Furthermore, adjusting the dosage regimens based on the *Candida* species and the corresponding MICs for both adult ICU and non-ICU patients should be considered. These findings provide valuable implications for dosing regimen optimization of MFG in critically ill patients.

## Figures and Tables

**Table 3:** Pharmacokinetic parameters of micafungin over 24 h at steady state for various groups.

	Neonates	Infants	Preschool Children	School Children	Adolescents	ICU Adults (SOFA ≥ 10)	ICU Adults (SOFA < 10)	Non-ICU Adults
BW-adjusted CL (mL/h/kg)	23.3 (17.2–32)	19.3 (16.2–22.9)	16.3 (13.7–19.4)	13.9 (11.6–16.7)	12.8 (10.3–15.6)	14.7 (10.7–19.7) ^a^	15.9 (10.7–20.1) ^a^	11.3 (9.5–15)
						15.9 (13–21.1) ^b^	17.1 (13.4–21.3) ^b^	
BW-adjusted Vd (mL/kg)	323.3 (238.4–445.3)	185.3 (115.4–258.7)	160.4 (98.5–219.1)	142.2 (81.7–196.1)	105.2 (48–158.6)	174.2 (133–226.5) ^a^	174.2 (134.9–243.5) ^a^	136.1 (96.2–162.3)
						183.9 (150.7–239.7) ^b^	187.4 (149.4–262.9) ^b^	
AUC_24_ (mg·h/L)	162.5 (116.9–222.2)	103 (86.9–122.6)	122.2 (102.6–145.4)	143.4 (119.7–171.5)	155.7 (127.8–194.1)	96.8 (72–132.3) ^a^	89.7 (70.7–131.7) ^a^	125.6 (95.3–149.4)
						89.8 (67.4–109.5) ^b^	83.2 (67–106) ^b^	

^a^ Results from all included studies; ^b^ Results from all included studies except Zhong et al. [29]. MFG was intravenously administered over 1 h once daily for 7 days to the below virtual groups: 4 mg/kg/d in neonates (14 days old, 1.5 kg), 2 mg/kg in infants (1 year old, 8 kg), preschool children (3 years old, 15 kg) and school children (7 years old, 30 kg), and 100 mg in adolescents (14 years old, 50 kg) and adults (40 years old, 70 kg); The SOFA score were set to 7 for ICU patients with SOFA < 10 and 11 for ICU patients with SOFA ≥ 10. Data are expressed as median values (25~75%) from all patients within each group.

**Table 4:** The minimum dosage to sustain given ratios of studies reaching optimal dosage criterion.

	50%		60%		70%		80%	
	ICU (mg/d)	Non-ICU (mg/d)	ICU (mg/d)	Non-ICU (mg/d)	ICU (mg/d)	Non-ICU (mg/d)	ICU (mg/d)	Non-ICU (mg/d)
*C. albican*	150	100	150	100	150	150	200	150
*C. glabrata*	250	200	300	250	300	250	>300	300
*C. krusei*	>300	>300	>300	>300	>300	>300	>300	>300
*C. tropicalis*	250	200	>300	200	>300	300	>300	>300
*C. parapsilosis*	>300	>300	>300	>300	>300	>300	>300	>300

50%, 60%, 70%, and 80% were ratios of studies reaching optimal dosage criterion; All patients were 70 kg and received MFG once daily for 7 days; The SOFA score in the ICU adults group was set to 11.

## Data Availability

The data generated during and/or analyzed during the current study are available from the published literature.

## Supplementary Materials

The following supporting information can be downloaded at https://www.mdpi.com/article/10.3390/pharmaceutics16091145/s1: Figure S1: Simulated concentration–time profiles of micafungin over 24 h at steady state; Figure S2: Distribution of the central volume of micafungin in various typical virtual populations over 24 h at steady state; Figure S3: The histogram of the amount of investigated and identified covariates in included studies; Figure S4: The probability target achievement of micafungin over 24 h at steady state for ICU adults or non-ICU adults against *C. Albicans*, *C. glabrata*, *C. Krusei*, *C. tropicalis*, and *C. parapsilosis* in included studies; Table S1: Demographic information of simulated patients; Table S2: Checklist for literature quality when reporting a clinical pharmacokinetic study; Table S3: List of tested and significant covariates in the included models; Table S4: The effect of covariates on the range of CL in each study; PRISMA 2020 Main Checklist.
