# Supplementary material for: Evaluation of Population Pharmacokinetic Models of Micafungin: Implications for Dosing Regimen Optimization in Critically Ill Patients
Source: Pharmaceutics. 2024 Aug 29;16(9):1145. doi: 10.3390/pharmaceutics16091145 (PMC11434802; doi:10.3390/pharmaceutics16091145)
Supplement: Supplementary file 1 [file pharmaceutics-16-01145-s001.zip › Supplementary figures and tables.pdf]

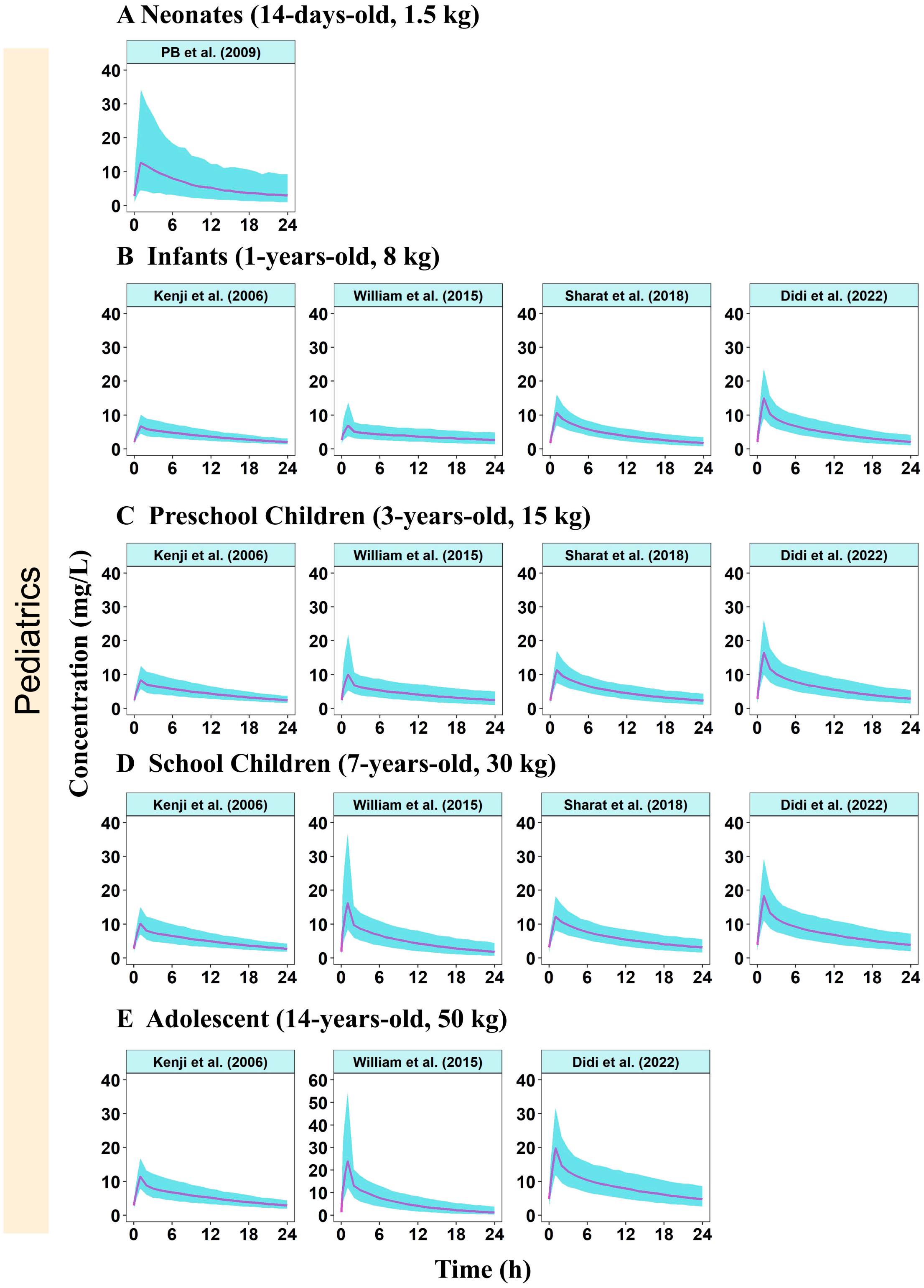

Figure S1. Simulated concentration-time profiles of micafungin over 24 h at steady state for (**A**) neonates, (**B**) infants, (**C**) preschool children, (**D**) school children and (**E**) adolescents [16,18-21]. The solid orange lines represent the median of the simulated concentration-time profiles and the light orange shadows represent the 5th–95th percentiles of the concentration-time profiles. MFG was intravenously administered once daily for 7 days as follows: 4 mg/kg in neonates, 2 mg/kg in children younger than 12-year-old, and 100 mg/day in adolescents and adults.

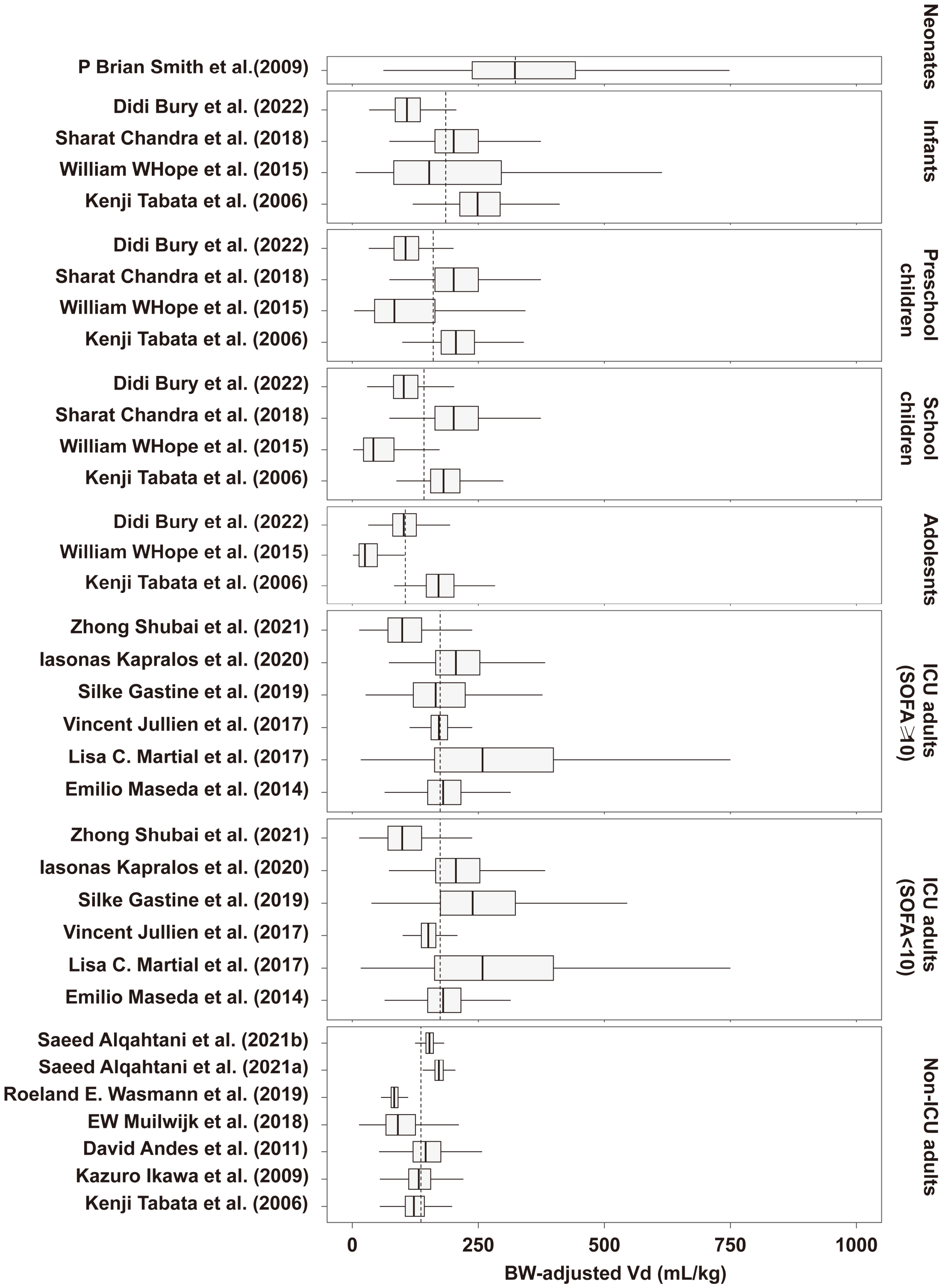

Figure S2. Distribution of the BW-adjusted Vd of micafungin in various typical virtual populations over 24 h at steady state [8,11,12,16–21,27–33]. The SOFA score were set to 7 for ICU patients with SOFA<10 and 11 for ICU patients with SOFA≥10; The dashed lines in each panel represented the median values of Vd per body weight from all patients within each group.

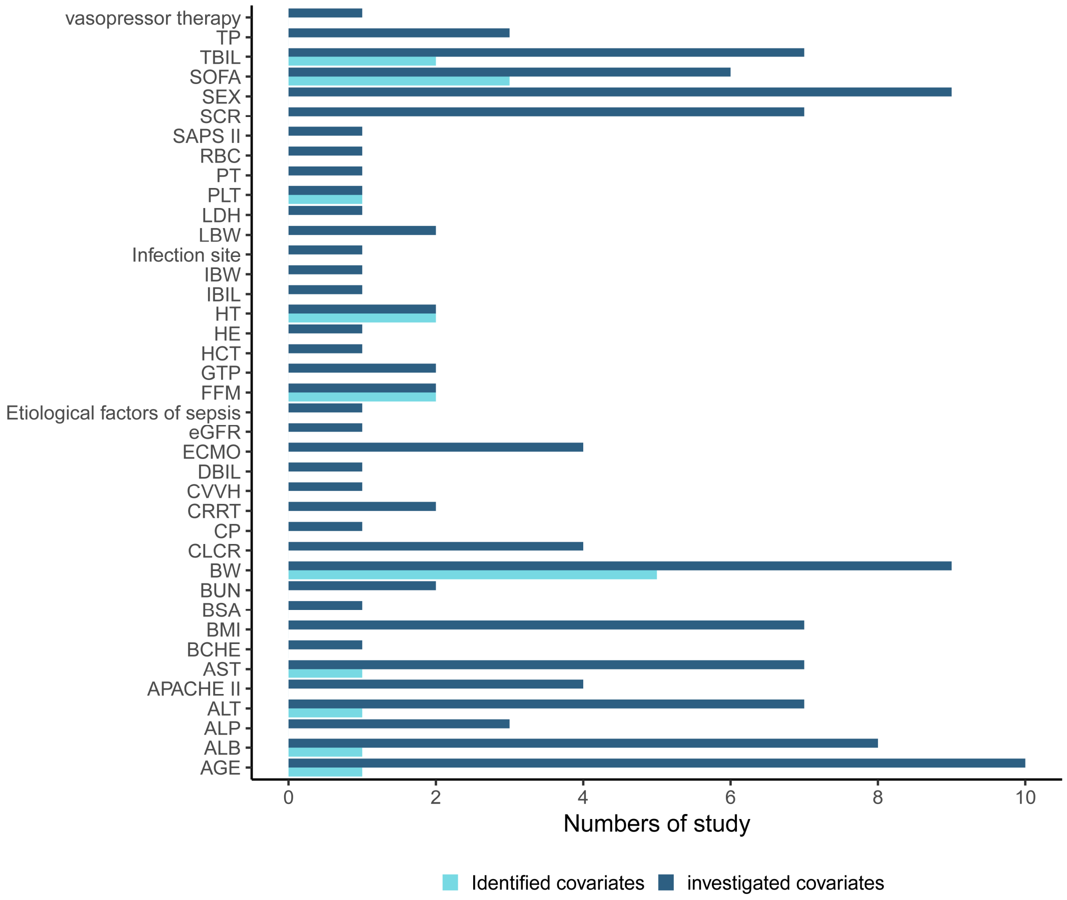

Figure S3. The histogram of the amount of investigated and identified covariates in included studies. AGE, age; HT, height; LBW, lean body weight; FFM, fat-free mass; IBW, idea body weight; TBW or BW, Total body weight; BSA, body surface area; BMI, body mass index; ALP, alkaline phosphatase; BCHE, butyrylcholinesterase; CVVH, Continuous Veno-Venous Hemofiltration; HE, haemodialysis; RBC, red blood cell count; HCT, hematocrit; PLT, platelet count; eGFR, estimated glomerular filtration rate; CLCR, creatinine clearance; SCR, serum creatinine; CRRT, continuous renal replacement therapy; TP, total protein; ALB, albumin; BUN, blood urea nitrogen; TBILI, total bilirubin; DBIL, direct bilirubin; IBIL, indirect bilirubin; ALT, alanine amino transferase or glutamic pyruvic oxaloacetate transaminase/aspartate aminotransferase; AST, aspartate aminotransferase; GTP, glutamate dehydrogenase; LDH, lactate dehydrogenase; SOFA, Sepsis-related organ failure assessment score, SAPS II, Simplifed Acute Physiology Score; CP, Child-Pugh score.

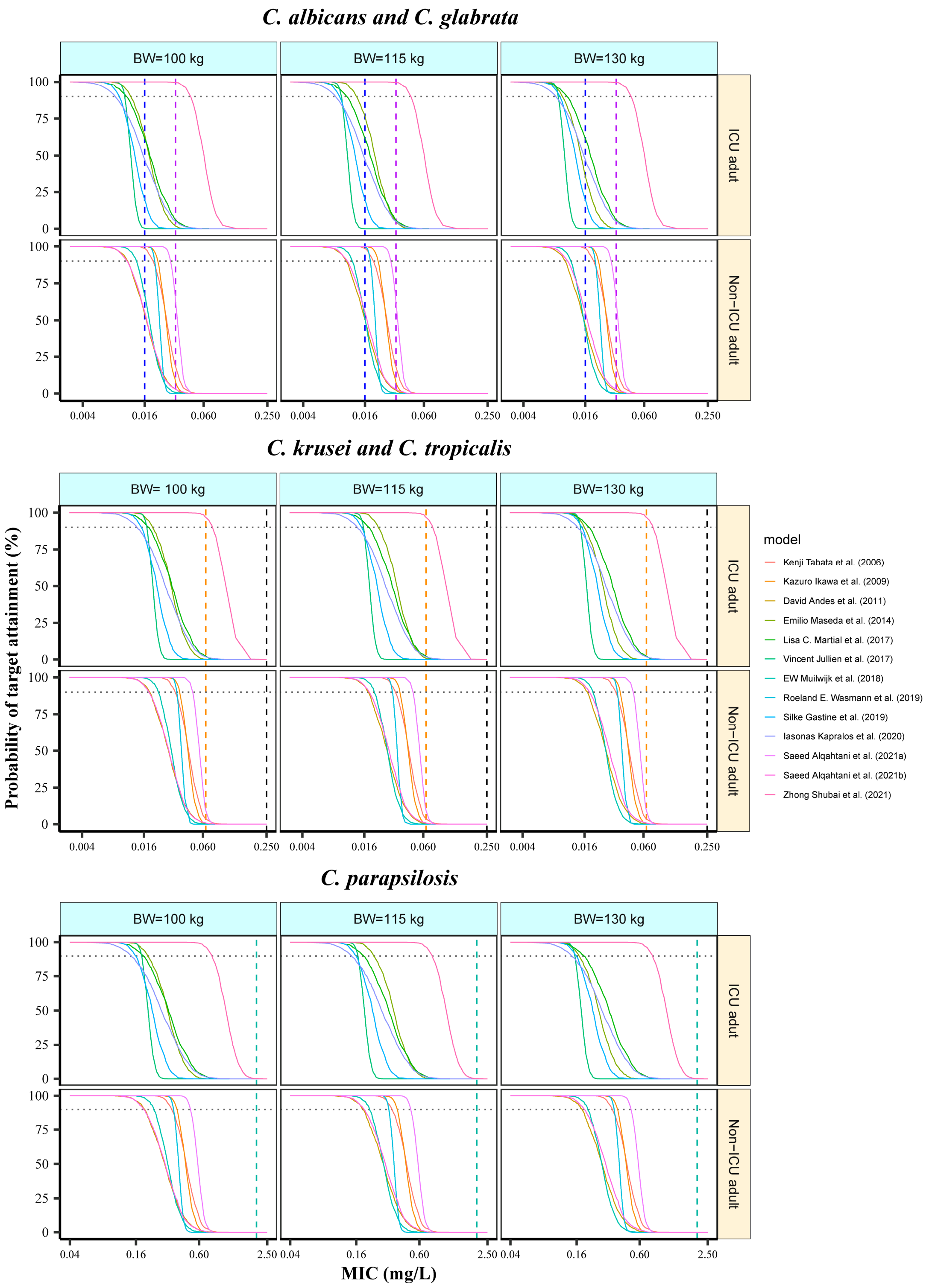

Figure S4. The probability target achievement of micafungin over 24h at steady state for ICU adults or Non-ICU adults against *C. Albicans*, *C. glabrata*, *C. Krusei*, *C. tropicalis* and *C. parapsilosis* in included studies [8,11,12,16,17,27–33]. The MIC breakpoints for *C. Albicans* (blue), *C. glabrata* (pink), *C. Krusei* (black), *C. tropicalis* (orange) and *C. parapsilosis* (green) are marked with dashed and dotted lines in each panel, respectively. A PTA of 90% is highlighted with black dashed lines.

Table S1. Demographic information of simulated patients.

		median	min	max
Adults				
	Sex	Male		
	Age (years)	40	20	80
	Body weight (kg)	70	40	150
	Fat-free weight (kg)	57.2	39.7	83.3
	Height (cm)	180	150	190
	Body mass index (kg/m^2)	20	18	30
	Platelet count (10^4/μL)	21.6	12.5	35
	Infusion rate (mg/h)	100		
	I Non-ICU			
	Alanine aminotransferase (U/L)	25	0	50
	Aspartate aminotransferase (U/L)	25	0	50
	Total bilirubin (μmol/L)	12	0	17.1
	Albumin (/min)	40	20	60
	Sepsis related organ failure assessment score	0	0	0
	II ICU			
	Alanine aminotransferase (U/L)	50	0	199
	Aspartate aminotransferase (U/L)	50	0	340
	Total bilirubin (μmol/L)	70	0	100
	Albumin (/min)	24	9	66
	Sepsis related organ failure assessment score	11	0	19
Children				
I	Neonate (≤1 month)			
	Sex	Male		
	Age (years)	0.04	0.003	0.08
	Body weight (kg)	1.5	0.5	2.5
	Fat-free weight (kg)	1.6	0.63	2.3
	Height (cm)	40	20	50
	Body mass index (kg/m^2)			
II	infants (4 months to 2 years)			
	Sex	Male		
	Age (years)	1.0	0.6	1.4
	Body weight (kg)	8	6	10
	Fat-free weight (kg)	7.79	6.31	9.06
	Height (cm)	78	60	96
	Body mass index (kg/m^2)	13	10	30
III	Preschool Children (2 to 6 years)			
	Sex	Male		
	Age (years)	3.7	2.5	4.9

	Body weight (kg)	15	10	20
	Fat-free weight (kg)	13.88	10.41	16.65
	Height (cm)	98.5	87	110
	Body mass index (kg/m²)	15	10	30
IV	School Children (6 to 12 years)			
	Sex	Male		
	Age (years)	9	8.5	10.5
	Body weight (kg)	30	20	40
	Fat-free weight (kg)	26	20.07	31.44
	Height (cm)	130	115	145
	Body mass index (kg/m²)	17	10	30
V	Adolescent (12 to 16 years)			
	Sex	Male		
	Age (years)	14.5	13	16
	Body weight (kg)	50	37	70
	Fat-free weight (kg)	42.53	34.99	51.56
	Height (cm)	160	150	170
	Body mass index (kg/m²)	19	10	30

Table S2. Checklist for literature quality when reporting a clinical pharmacokinetic study.

	Item	Kenji Tabata et al. (2006)	Kazuro Ikawa et al. (2009)	P Brian Smith et al. (2009)	David Andes et al. (2011)	Emilio Maseda et al. (2014)	William W. Hope et al. (2015)	Lisa C. Martial et al. (2017)	Vincent Jullien et al. (2017)	E. W. Muilwijk et al. (2018)	Sharat Chandra et al. (2018)	Roeland E. Wasmann et al. (2019)	Silke Gastine et al. (2019)	Zhong Shubai et al. (2019)	Iasonas Kapralos et al. (2020)	Saeed Alqahtani et al. (2021a)	Saeed Alqahtani et al. (2021b)
1	Drug and patient population	1	1	1	1	1	1	1	1	1	1	1	1	1	1	1	1
2	The name of the drug(s) studied, the route of administration, the population in whom it was studied, and the results of the primary objective and major clinical pharmacokinetic findings	1	1	1	1	1	1	1	1	1	1	1	1	1	1	1	1
3	Pharmacokinetic data	1	1	1	1	1	1	1	1	1	1	1	1	1	1	1	1
4	An explanation of the study rationale	1	1	1	1	1	1	1	1	1	1	1	1	1	1	1	1
5	Specific objectives or hypotheses	1	1	1	1	1	1	1	1	1	1	1	1	1	1	1	1
6	Eligibility criteria of study participants	1	1	1	1	1	1	1	1	1	1	1	1	1	1	1	1
7	Co-administration with other potentially interacting drugs or food	0	0	0	0	1	1	1	0	1	0	0	0	0	0	0	0

	Item	Kenji Tabata et al. (2006)	Kazuro Ikawa et al. (2009)	P Brian Smith et al. (2009)	David Andes et al. (2011)	Emilio Maseda et al. (2014)	William W. Hope et al. (2015)	Lisa C. Martial et al. (2017)	Vincent Jullien et al. (2017)	E. W. Muilwijk et al. (2018)	Sharat Chandra et al. (2018)	Roeland E. Wasmann et al. (2019)	Silke Gastine et al. (2019)	Zhong Shubai et al. (2019)	Iasonas Kapralos et al. (2020)	Saeed Alqahtani et al. (2021a)	Saeed Alqahtani et al. (2021b)
8	Drug preparation and administration characteristics including dose, route, formulation, infusion duration (if applicable) and frequency	1	1	1	1	1	1	1	1	1	1	1	1	1	1	1	1
9	Body fluid or tissue sampling (timing, frequency and storage) for quantitative drug measurement	1	1	1	1	1	1	1	1	1	1	1	1	1	1	1	1
10	Quantitative bioanalytical methods	1	1	1	0	1	1	1	1	1	1	1	1	1	1	1	1
11	Methods for handling missing data	1	0	0	0	0	1	1	1	0	0	0	0	0	0	0	0
12	Modelling software	1	1	1	1	1	1	1	1	1	1	1	1	1	1	1	1
13	Statistical methods and software	1	1	1	1	1	1	1	1	1	1	1	1	1	1	1	1
14	Candidate strutural models	1	1	1	1	1	1	1	1	1	0	1	1	1	1	1	1
15	Residual error struture	1	1	1	1	1	1	1	1	1	1	1	1	1	1	1	1
16	Methods for base model determination	1	1	1	1	1	1	1	1	1	1	1	1	1	1	1	1

	Item	Kenji Tabata et al. (2006)	Kazuro Ikawa et al. (2009)	P Brian Smith et al. (2009)	David Andes et al. (2011)	Emilio Maseda et al. (2014)	William W. Hope et al. (2015)	Lisa C. Martial et al. (2017)	Vincent Jullien et al. (2017)	E. W. Muilwijk et al. (2018)	Sharat Chandra et al. (2018)	Roeland E. Wasmann et al. (2019)	Silke Gastine et al. (2019)	Zhong Shubai et al. (2019)	Iasonas Kapralos et al. (2020)	Saeed Alqahtani et al. (2021a)	Saeed Alqahtani et al. (2021b)
17	Methods for base model evaluation	1	1	1	0	1	1	1	1	1	1	1	1	1	1	1	1
18	Covariates analysis strategy	1	0	0	1	1	1	1	1	1	1	1	1	1	1	1	1
19	Methods for final model evaluation	1	1	1	1	1	1	1	1	1	1	1	1	1	1	1	1
20	Distribution of individual model parameters	0	1	1	1	1	1	1	1	1	1	1	1	1	1	1	1
21	Estimation methods	1	1	1	1	1	1	1	1	1	1	1	1	1	1	1	1
22	Population characteristics	1	1	1	1	1	1	1	1	1	1	1	1	1	1	1	1
23	Schematic of the final model	0	0	0	0	0	1	0	0	0	0	0	0	0	0	0	0
24	Number of subjects and observations	1	1	1	1	1	1	1	1	1	1	1	1	1	1	1	1
25	Table of the final model parameter	1	1	1	1	1	1	1	1	1	1	1	1	1	1	1	1
26	Final model evaluation plots	1	1	0	0	1	1	1	1	1	1	1	1	1	1	1	1
27	Summary of the model-building process and the derived final model	1	0	0	1	1	1	1	1	1	0	1	1	1	1	1	1

	Item	Kenji Tabata et al. (2006)	Kazuro Ikawa et al. (2009)	P Brian Smith et al. (2009)	David Andes et al. (2011)	Emilio Maseda et al. (2014)	William W. Hope et al. (2015)	Lisa C. Martial et al. (2017)	Vincent Jullien et al. (2017)	E. W. Muilwijk et al. (2018)	Sharat Chandra et al. (2018)	Roeland E. Wasmann et al. (2019)	Silke Gastine et al. (2019)	Zhong Shubai et al. (2019)	Iasonas Kapralos et al. (2020)	Saeed Alqahtani et al. (2021a)	Saeed Alqahtani et al. (2021b)
28	Plot of concentration versus times and/or effect versus concentration	1	0	1	0	1	1	1	0	0	1	1	1	1	0	0	0
29	Study limitation	0	1	1	1	1	1	1	1	1	1	1	1	1	1	1	1
30	Study findings	1	1	1	1	1	1	1	1	1	1	1	1	1	1	1	1
	Total score	26	24	24	23	28	30	29	27	27	25	27	27	27	26	26	26
	**Compliance**	**86.67%**	**80%**	**80%**	**76.67%**	**93.33%**	**100%**	**96.67%**	**90%**	**90%**	**83.33%**	**90%**	**90%**	**90%**	**86.67%**	**86.67%**	**86.67%**

Table S3. List of tested and significant covariates in the included models.

Study (publication year)	Tested covariates			Covariate selection		Significant covariates	
	Demographic	Laboratory tests	Co-administration	Forward inclusion	Backward elimination	*CL*	*V*
Kenji Tabata (2006)	SEX, AGE, WT, BMI	RBC, HCT, PLT, TP, ALB, BUN, TBIL, ALT, AST, γ-GTP, LDH, ALP, CRCl, SCR,	NR	$p < 0.05$	$p < 0.001$	AGE, WT, PLT	NR
Kazuro Ikawa (2009)	NR	NR	NR	$p < 0.05$	$p < 0.05$	NR	NR
P Brian Smith (2009)	NR	NR	NR	$P < 0.05$	$P < 0.05$	NR	NR
David Andes (2011)	BW	NR	NR	$p < 0.05$	$p < 0.001$	BW	NR
Emilio Maseda (2014)	AGE, SEX, BW	TBIL, SCR, BUN	vasopressor therapy	$p < 0.05$	$p < 0.05$	BW	NR
William W. Hope (2015)	AGE, SEX	ALB, PLT, RBC, SCR, ALT, AST, TBIL	NR	$p < 0.05$	$p < 0.01$	BW, AST, TBIL	BW
Lisa C. Martial (2017)	NR	ALB, CVVH, SOFA	NR	$p < 0.05$	$p < 0.05$	NR	NR
Vincent Jullien (2017)	AGE, SEX, BW	TP, ALT, AST, ALP, ALB, TBIL, PT, SOFA, ECMO, HE	NR	$p < 0.05$	$p < 0.01$	BW, ALB, SOFA	BW, ALB
E.W. Muilwijk (2018)	FFM	NR	NR	$P < 0.05$	$p < 0.05$	FFM	FFM
Sharat Chandra (2018)	AGE, SEX, BW, BMI	APACHE II, SCR, CRCl, ALB	NR	$P < 0.05$	$p < 0.01$	BW	BW
Roeland E. Wasmann (2019)	SEX, AGE, TBW, LBW, IBW, BMI	NR	NR	$p < 0.05$	$P < 0.001$	TBW	TBW
Silke Gastine (2019)	SEX, WT, AGE, BMI	ALB, TP, CLCR, ALT, AST, BCHE, TBIL, APACHE II, SOFA, SAPS II	NR	$p < 0.05$	$p < 0.01$	TBIL	SOFA
Iasonas Kapralos (2020)	AGE, BW, HT,BSA, LBW, BWI	ALB, PLT, HCT, RBC, SCR, eGFR, ALT, AST, γ-GTP, AST, ALT, GGT, ALP, TBIL, SOFA, APACHE II, CRRT	NR	$p < 0.05$	$p < 0.01$	NR	NR

Study (publication year)	Tested covariates			Covariate selection		Significant covariates	
	**Demographic**	**Laboratory tests**	**Co-administration**	**Forward inclusion**	**Backward elimination**	***CL***	***V***
Saeed Alqahtani (2021)	SEX, AGE, BW, HT, BMI	SCR, CRCl, SOFA, AST, AST, TBIL, ALB	NR	$p < 0.05$	$p < 0.05$	NR	NR
Zhong Shubai (2021)	SEX, AGE, BW, BMI	ALT, AST, TP, SCR, TP, DBIL, IBIL, etiological factors of sepsis, infection site, CP, APACHE II, SOFA, CRRT	NR	$p < 0.05$	$p < 0.001$	ALT	SOFA
Didi Bury (2022)	FFM	FFM	NR	$p < 0.05$	$p < 0.05$	FFM	FFM

SEX, sex; AGE, age; HT, height; TBW or BW, total body weight; LBW, lean body weight; FFM, fat-free mass; IBW, idea body weight; BW, body weight; WT, weight; BSA, body surface area; BMI, body mass index; CVVH, Continuous Veno-Venous Hemofiltration; HE, haemodialysis; RBC, red blood cell count; HCT, hematocrit; PLT, platelet count; eGFR, estimated glomerular filtration rate; CL, total clearance; CLCR, creatinine clearance; SCR, serum creatinine; CRRT, continuous renal replacement therapy; TP, total protein; ALB, albumin; BUN, blood urea nitrogen; TBILI, total bilirubin; DBIL, direct bilirubin; IBIL, indirect bilirubin; ALT, alanine amino transferase or glutamic pyruvic oxaloacetate transaminase/aspartate aminotransferase; AST, aspartate aminotransferase;γ-GTP, glutamate dehydrogenase; LDH, lactate dehydrogenase; ALP, alkaline phosphatase; BCHE, butyrylcholinesterase, APACHE II, Acute Physiology and Chronic Health Evaluation II; SOFA, Sepsis-related organ failure assessment score, SAPS II, Simplifed Acute Physiology Score; CP, Child-Pugh score; Q, intercompartmental (central-peripheral) clearance; V, volume of distribution; V_c, volume of distribution of the central compartment; V_p, volume of distribution of the central compartment.

Table S4. The effect of covariates on the range of CL in each study.

Study	Covariates	Range of Covariates		Reference value of CL	Range of CL (Ratio)	
		Minimum	Maximum		Minimum	Maximum
Kenji Tabata *et al*. (2006)	Weight (kg)	6	10	0.17	0.14 (0.84)	0.18 (1.06)
William W. Hope *et al*. (2015)		6	10	0.16	0.13 (0.82)	0.19 (1.18)
Sharat Chandra *et al*. (2018)		6	10	0.15	0.12 (0.80)	0.18 (1.17)
Didi Bury *et al*. (2022) *		6	10	0.13	0.11 (0.85)	0.15 (1.12)
Kenji Tabata *et al*. (2006)		10	20	0.27	0.19 (0.72)	0.33 (1.22)
William W. Hope *et al*. (2015)		10	20	0.27	0.20 (0.75)	0.32 (1.19)
Sharat Chandra *et al*. (2018)		10	20	0.25	0.17 (0.71)	0.29 (1.20)
Didi Bury *et al*. (2022) *		10	20	0.25	0.20 (0.81)	0.28 (1.15)
Kenji Tabata *et al*. (2006)		20	40	0.47	0.39 (0.83)	0.71 (1.51)
William W. Hope *et al*. (2015)		20	40	0.48	0.40 (0.83)	0.67 (1.39)
Sharat Chandra *et al*. (2018)		20	40	0.41	0.35 (0.85)	0.59 (1.42)
Didi Bury *et al*. (2022) *		20	40	0.38	0.34 (0.81)	0.43 (1.14)
Kenji Tabata *et al*. (2006)		37	70	0.78	0.57 (0.73)	1.02 (1.31)
William W. Hope *et al*. (2015)		37	70	0.69	0.55 (0.79)	0.92 (1.33)
Didi Bury *et al*. (2022) *		37	70	0.54	0.47 (0.86)	0.63 (1.16)
Kenji Tabata *et al*. (2006)		40	150	0.78	0.78 (1)	0.78 (1)
David Andes *et al*. (2011)		40	150	1.07	0.93 (0.87)	1.30 (1.22)
Emilio Maseda *et al*. (2014)		40	150	0.88	0.58 (0.66)	1.56 (1.77)
Vincent Jullien *et al*. (2017)		40	150	1.20	0.96 (0.80)	1.82 (1.51)
E. W. Muilwijk *et al*. (2018) *		40	150	1.01	0.77 (0.76)	1.34 (1.33)
Roeland E. *et al*. (2019)		40	150	0.69	0.46 (0.66)	1.21 (1.76)
Kenji Tabata *et al*. (2006)	Children: PLT (10^4/μL)	1.8	48.6	0.16	0.14 (0.91)	0.18 (1.15)
Kenji Tabata *et al*. (2006)	Adult: PLT (10^4/μL)	0.3	74.5	0.47	0.45 (0.95)	0.54 (1.14)
Zhong Shubai *et al*. (2021)	ALT (U/L)	8	200	0.58	0.22 (0.38)	0.72 (1.24)
William W. Hope *et al*. (2015)	AST (U/L)	3	340	0.48	0.41 (0.85）	0.55 (1.14)
William W. Hope *et al*. (2015)	TBIL (μmol/L)	4.5	42.2	0.46	0.43 (0.94)	0.49 (1.05)
Silke Gastine *et al*. (2019) **	TBIL ≤68.4 (μmol/L)	0	1	1.56	1.23 (0.79)	1.56 (1.00)
Vincent Jullien *et al*. (2017) **	ALB ≤25 (g/L)	0	1	1.37	1.37 (1.00)	1.56 (1.14)
Vincent Jullien *et al*. (2017) **	SOFA ≥ 10	0	1	1.37	1.03 (0.75)	1.37 (1.00)

* FFM was transformed to equivalent BW; ** For binary covariates, SOFA, 0 for SOFA ≥10 and 1 for SOFA <10; TBIL, 0 for TBIL ≥ 68.4 μmol/L and 1 for TBIL <68.4 μmol/L; ALB, 0 for ALB ≤25 (g/L) and ALB >25 (g/L); BW, body weight; FFM, free-fat mass; ALT, alanine amino transferase; AST, aspartate aminotransferase; PLT, platelet count; TBILI, total bilirubin; ALB, albumin; SOFA, Sepsis-related organ failure assessment score.
